# Boosted H_2_O_2_ Photosynthesis via Synergistic Tuning Electronic Structure and Interfacial O_2_ Transfer on Floatable Carbon Nitride Networks

**DOI:** 10.3390/molecules31203770

**Published:** 2026-10-20

**Authors:** Xiaoying Peng, Zhangsong Jiang, Fanghua Liu, Bingzhi Xiang, Jielu Zhu, Jie Lin, Bo Wang, Junwei Song, Kai Yang, Guiming Peng

**Affiliations:** 1 Research Center of Advanced Materials and Green Energy, Jiangxi Institute of Technology, Nanchang 330098, China; xypeng0810@163.com (X.P.);; 2 School of Chemistry and Chemical Engineering, Jiangxi University of Science and Technology, Ganzhou 341000, China; 3 Key Laboratory of Fluorine and Silicon for Energy Materials and Chemistry of Ministry of Education, College of Chemistry and Materials, Jiangxi Normal University, Nanchang 330022, China

**Keywords:** carbon nitride, H_2_O_2_, photosynthesis, triphase interface

## Abstract

Solar-driven photosynthesis of hydrogen peroxide (H_2_O_2_) from O_2_ and H_2_O offers an eco-friendly and sustainable route. Metal-free graphitic carbon nitride (CN) is capable of photocatalytic H_2_O_2_ production, yet its efficiency is limited by insufficient charge separation and slow O_2_ supply in conventional diphase systems. Herein, we graft cinnamylidene groups into CN to fabricate a hydrophobic CN nanolayer (CNCA/CF). The incorporated cinnamylidene groups with extended conjugated chains narrow the bandgap, promote photocarrier separation and improve 2e ORR selectivity. Benefiting from the hydrophobicity of CNCA/CF, it can stably float on water and construct a gas–solid–liquid triphase interface that enables rapid O_2_ diffusion and delivery. Moreover, floating CNCA/CF exhibits a high H_2_O_2_ output of 1370 μmol m^−2^ h^−1^ in a homemade reactor, 15.3-fold higher than that of pristine CN. Mechanistic studies demonstrated that H_2_O_2_ is generated through a sequential 2e ORR process involving ·O_2_^−^ as the crucial intermediate. This work highlights a feasible strategy combining triphase interface optimization with electronic tuning, providing a new platform for the rational design of photocatalysts for H_2_O_2_ triphase photosynthesis.

## 1. Introduction

As a sustainable and multifunctional chemical, hydrogen peroxide (H_2_O_2_) is widely utilized in diverse fields such as chemical production, medicine, textiles and clean fuels [1,2]. Currently, the anthraquinone oxidation process remains the prevailing technology for commercial H_2_O_2_ manufacture, while it suffers from various disadvantages, including complex reaction pathways, high energy consumption and toxic by-products [3]. Therefore, photocatalytic oxygen reduction reaction (ORR) has drawn considerable attention, as it generates photogenerated electrons under solar illumination, which can reduce dissolved oxygen in water and achieve solar-to-H_2_O_2_ conversion [4,5,6]. To date, various photocatalysts, ranging from metal-based photocatalysts [7,8] to organic polymeric materials [9,10] have been employed for H_2_O_2_ photogeneration. Among them, graphitic carbon nitride (CN) shows great promise for H_2_O_2_ production, benefiting from suitable bandgap structure, easy availability and facile modification [11,12,13]. However, photocatalytic H_2_O_2_ evolution via ORR over CN is still hindered by inherent shortcomings, such as the narrow absorption range, sluggish carrier migration and insufficient active sites [14,15,16]. Consequently, to address these issues, extensive investigations have been focused on modifying CN to boost H_2_O_2_ production [17,18,19]. Among these approaches, terminal conjugated modification mitigates edge electron localization and enhances in-plane electron delocalization, which improves carrier transport and light absorption [20]. Surface functionalization with conjugated chains is regarded as a promising route for enhancing light absorption and prompting surface charge migration of CN [21].

For conventional diphase systems, the oxygen involved in photocatalytic ORR primarily relies on dissolved oxygen in the aqueous phase [22]. The inherently low aqueous solubility of O_2_, coupled with its sluggish mass-transfer kinetics in water, restricts oxygen concentration at the catalyst interface, constituting a critical bottleneck for the kinetics of photocatalytic H_2_O_2_ synthesis [23]. These drawbacks reveal that, besides the design of the photocatalysts, the interfacial O_2_ supply plays an important role in promoting H_2_O_2_ generation [24]. However, few studies have explored the perspective of system design. Notably, in a gas–solid–liquid triphase interface, the rate of O_2_ delivery to the catalytic interface is approximately 10^4^ times relative to the liquid–solid diphase [25]. Thereby, the construction of a triphase interface can alleviate the sluggish O_2_ supply in the diphase and ensure adequate reactants for catalytic active sites. In recent years, most endeavors have employed materials to construct a photocatalytic triphase interface on hydrophobic porous substrates to accelerate oxygen transfer [23,24,25,26], significantly boosting H_2_O_2_ generation compared with diphase systems. However, most of these strategies rely on coating the catalyst onto the substrate surface, which usually results in weak catalyst–substrate interfacial adhesion. Such inferior interfacial bonding results in catalyst exfoliation during the reaction process and further adversely affects the long-term stability. Floatable catalysts can directly float on the surface of the liquid phase, which favor mass transfer of gaseous reactants. Hence, there is an urgent need for a facile design of in-situ self-floating photocatalysts to overcome these challenges by constructing stable triphase interfaces.

In this study, cinnamaldehyde was used to modify CN through a Schiff base reaction to form cinnamylidene groups, endowing the surface with hydrophobicity (denoted as CNCA/CF). The extended conjugated skeleton of grafted cinnamylidene moieties simultaneously tunes the electronic structure of CN. Benefiting from the inherent benzene ring and C=C double bond in cinnamylidene groups, this modification effectively broadens the light absorption range and promotes charge separation, while generating a stable hydrophobic surface. By floating on the water surface, CNCA/CF readily forms the gas–solid–liquid triphase interface for photocatalytic H_2_O_2_ production. The triphase system achieved a photocatalytic H_2_O_2_ production of 1370 μmol m^−2^ h^−1^ in pure water, corresponding to 6.2 and 15.3-fold enhancements over the diphase CNCA/CF and pristine CN, respectively. Experimental investigations proved a sequential two-step 1e ORR process. Mechanistic studies revealed that the unique hydrophobic surface and the contribution of cinnamylidene moieties are responsible for the superior photocatalytic performance.

## 2. Results and Discussion

**Figure 1:**
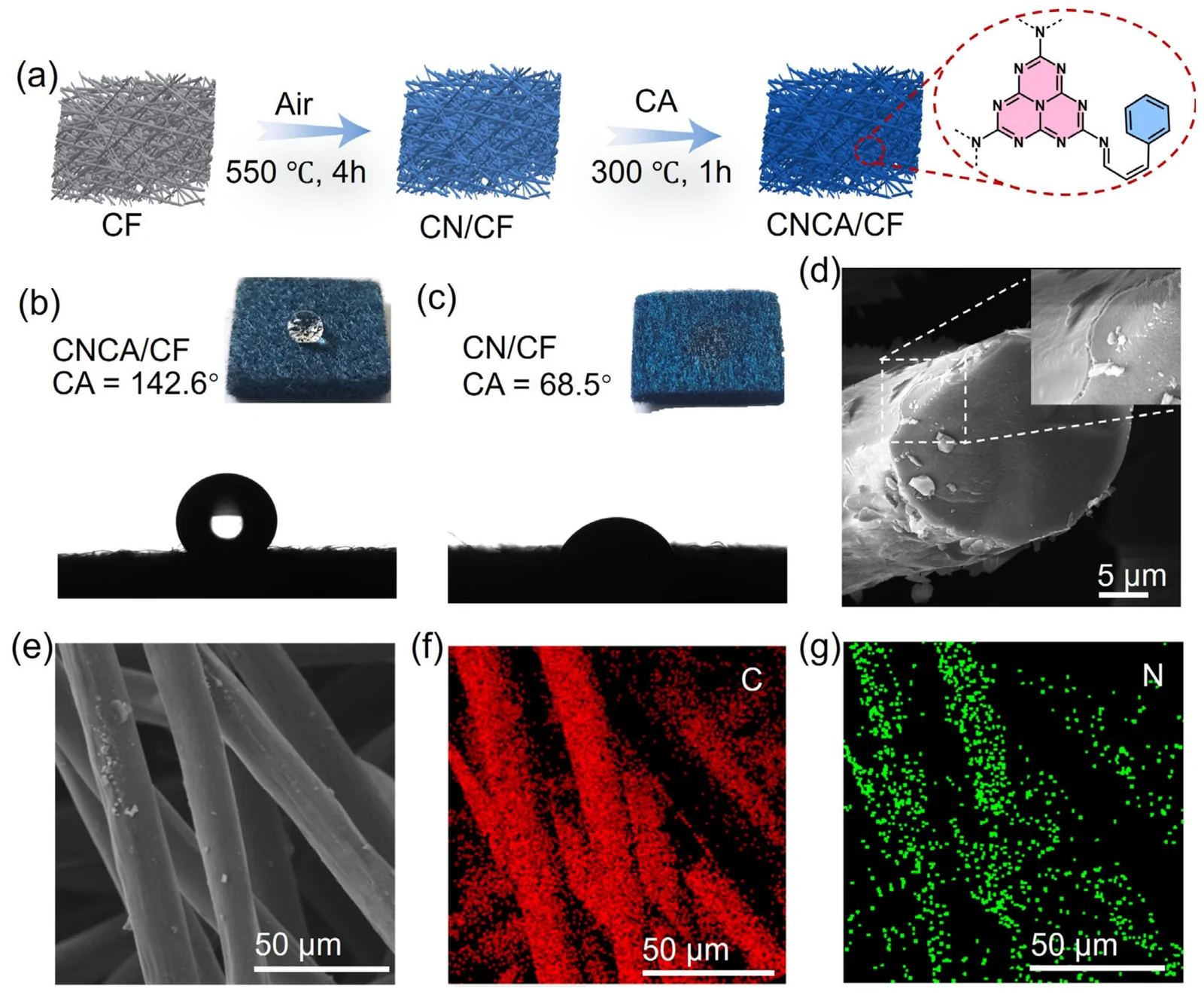
(**a**) Schematic preparation of CNCA/CF via chemical vapor deposition. Contact angle of (**b**) CNCA/CF and (**c**) CN/CF (inset are photographs of water droplets placed over CNCA/CF and CN/CF, respectively). (**d**) Cross-sectional and (**e**) top view SEM images of CNCA/CF. (**f**,**g**) EDX mapping images of C and N for CNCA/CF.

As depicted in Figure 1a, porous carbon nitride networks (CN/CF) were fabricated by in-situ growth of CN nanolayers on carbon felt (CF), following previously reported literature [27] (for the detailed process, see Supporting Information (SI)). Subsequently, a facile surface chemical modification rendered the CN nanolayer hydrophobic. This process proceeded via a Schiff base reaction between the aldehyde moieties of cinnamaldehyde (CA) and the terminal -NH_2_ groups of the CN framework (Figure S1). The material was denoted as CNCA/CF. The CN nanolayer maintains intimate contact with the carbon fiber substrate by means of the robust π–π interaction, leading to the blue color observed in both CNCA/CF and CN/CF in contrast to bare black CF (Figure 1b,c and Figure S2). Owing to the grafted cinnamylidene groups with benzene rings, CNCA/CF exhibits a water contact angle of 142.6° (Figure 1b), demonstrating the hydrophobicity of CNCA/CF. Thanks to a surface hydrophobicity of CNCA/CF, it achieves stable floating on the water surface (Figure S3a). Such a floatable CNCA/CF, with an oxygen saturated environment on its upper surface, facilitates the construction of a gas–solid–liquid triphase system, which also favors the diffusion of oxygen across the porous hydrophobic carbon fiber. On the contrary, the water contact angle of CN/CF is only 68.5° (Figure 1c), exhibiting hydrophilicity; therefore, it sinks at the bottom in water (Figure S3b). CN/CF is surrounded by water molecules, forming a diphase system. Accordingly, this triphase interface of CNCA/CF is expected to facilitate efficient H_2_O_2_ evolution by addressing the mass transfer of reactants. As clearly shown in Figure 1d, the CF substrate was uniformly coated with a CN nanolayer, which remains smooth as CF (Figure S4). Moreover, the top-view scanning electron microscopy (SEM) image and EDS elemental mapping reveals that the C elemental in the CN layer is uniformly distributed over the fiber (Figure 1e,f). EDS mapping of N further supports this uniform distribution, providing evidence for the successful fabrication of the CN nanolayer (Figure 1g and Figure S5).

**Figure 2:**
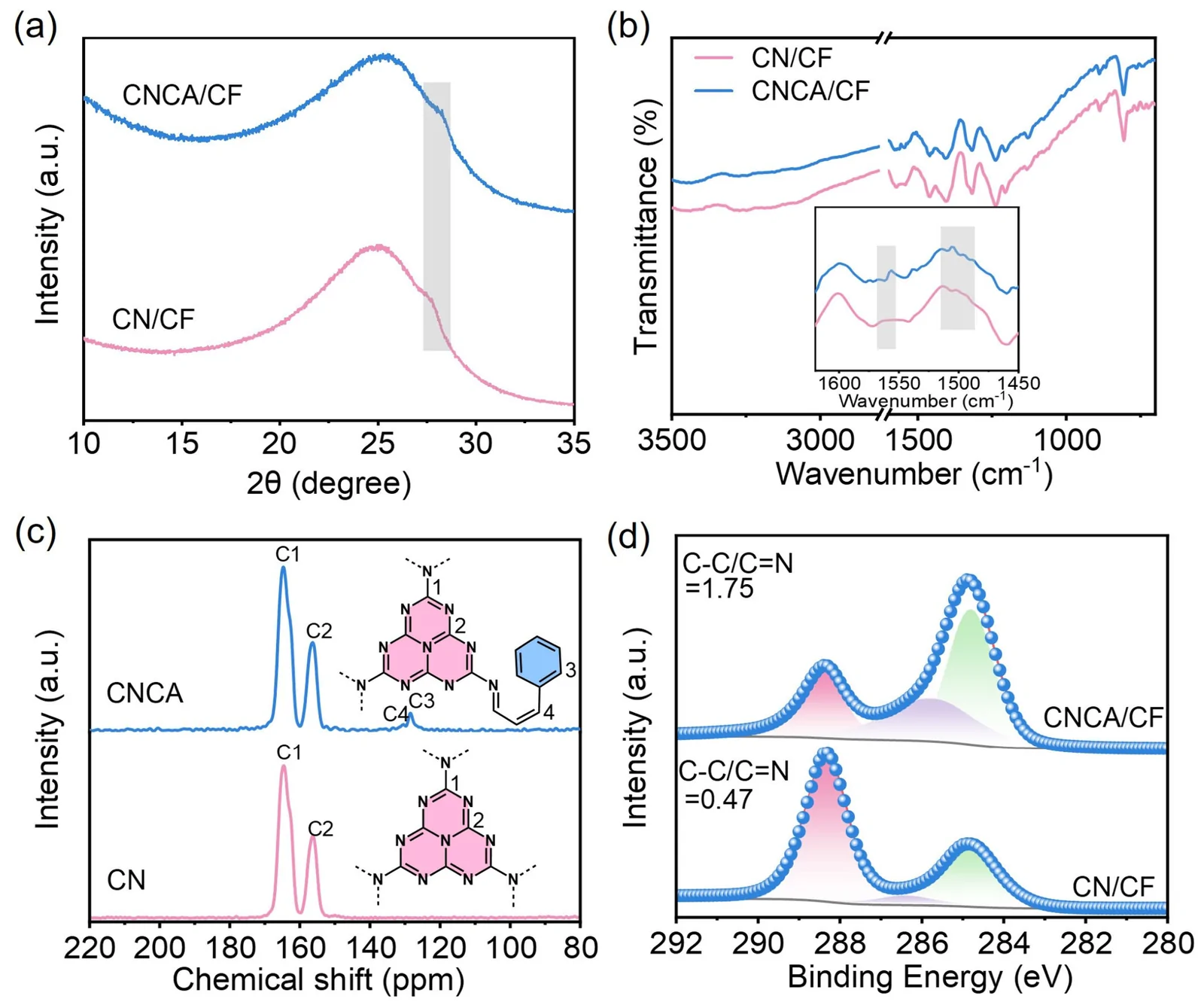
(**a**) XRD and (**b**) FTIR spectrum of CN/CF and CNCA/CF. (**c**) Solid-state ^13^C NMR spectra of CN and CNCA. (**d**) The C 1s XPS spectra of CN/CF and CNCA/CF.

In Figure 2a, a unique X-ray diffraction (XRD) peak of CN/CF and CNCA/CF is observed at 27.9° (002) relative to pristine CF, corresponding to the interlayer stacking of the CN nanolayer (Figure S6a) [28]. It shows a slight shift toward a higher angle relative to that of CN powder (27.5°, Figure S6b), suggesting a more compact interlayer stacking of the CN nanosheets. The characteristic absorption peak was measured by Fourier-transform infrared (FTIR) spectra. FTIR spectra exhibited the C–N heterocycle stretching vibrations in the range of 1200 to 1700 cm^−1^ (Figure 2b and Figure S6c) [29]. Additionally, a distinct band around 806 cm^−1^ corresponding to triazine moieties vibrations originated from the CN framework [30]. Notably, compared to CN/CF, two additional weak peaks for CNCA/CF were found around 1560 and 1499 cm^−1^, which arise from the skeletal vibrations of the phenyl ring on the cinnamylidene moieties [31,32]. This demonstrates that the cinnamylidene moieties were successfully grafted onto the CN nanolayer. Consistently, solid-state ^13^C nuclear magnetic resonance (NMR) spectra reveal that there are two prominent signals at 164.5 and 156.4 ppm for both CN/CF and CNCA/CF, which are identified as CN_2_-NH_x_ (C1) and −CN_3_ (C2) in the CN matrix, respectively (Figure 2c). Another two new peaks at 128.4 and 130.2 ppm are observed in CNCA/CF, which are assigned to the benzene carbon (C3) and C=C (C4) of the surface functionalities [33]. Besides, the XPS survey displays that the C/N ratio of CNCA/CF (3.07) is higher than that of CN/CF (0.96, Figure S7), further demonstrating the occurrence of a Schiff base reaction. Three distinct peaks at 284.8 eV, 288.4 and 285.8 were fitted in the C 1s spectrum, which were associated with C–C/C=C, C=N and C–NH_x_, respectively (Figure 2d) [19]. It is worth mentioning that CNCA/CF shows a significantly higher C–C/C=C peak intensity compared to CN/CF, which originates from the extended conjugated structure of cinnamylidene groups (Figure 2d). As for the N 1s spectrum, peaks constituents at 398.8, 400.5 and 401.3 eV due to the vibrations of C=N–C, N–C_3_ and C–NH_x_, respectively (Figure S7a) [29]. These findings suggest that cinnamylidene groups were successfully modified onto the CN framework after Schiff base formation. Collectively, the morphological, chemical and compositional analysis confirm that CN nanolayers were successfully coated and subsequently functionalized with cinnamylidene groups.

**Figure 3:**
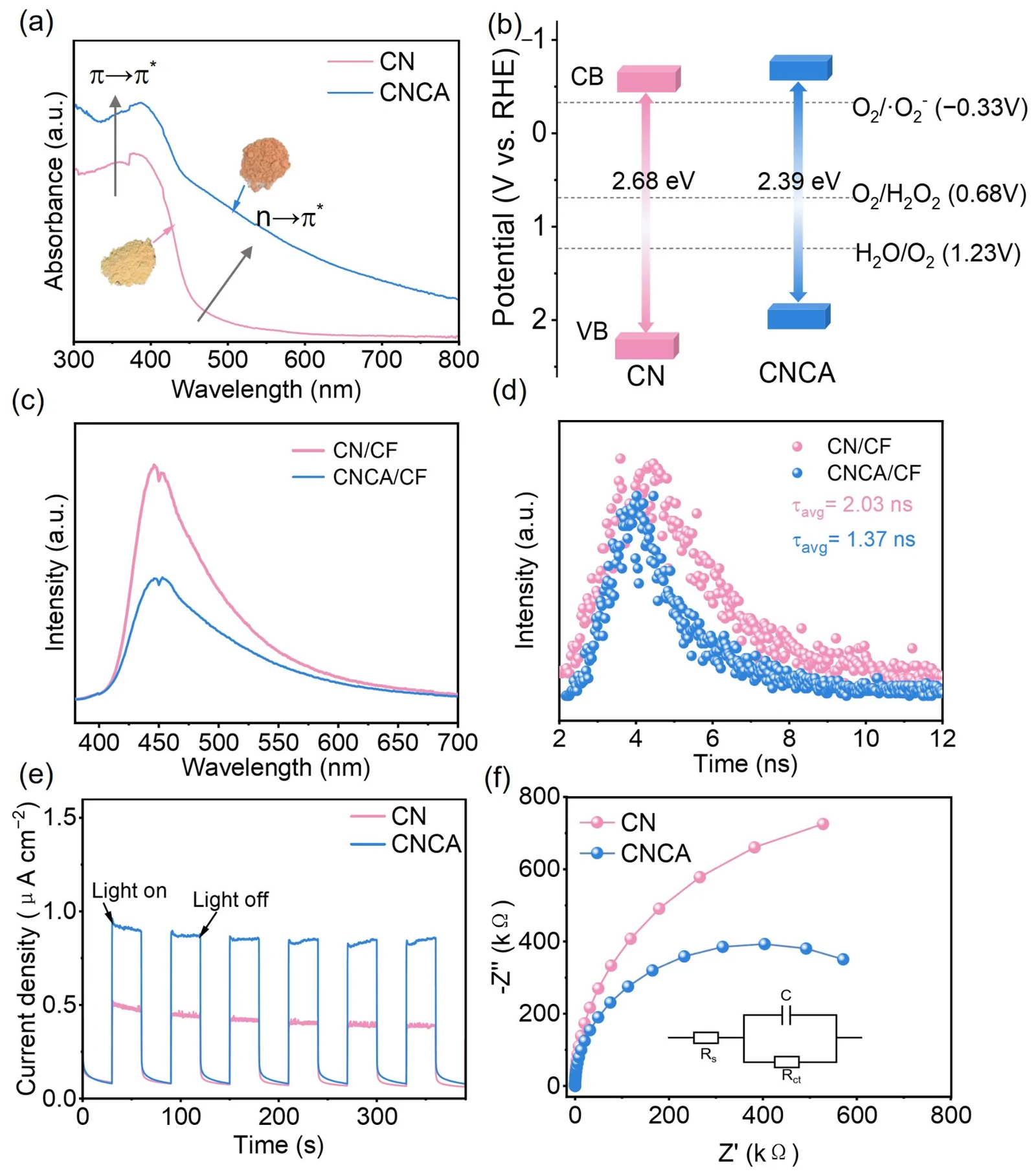
(**a**) UV–vis DRS (insets are photographs of CN and CNCA powder). (**b**) Band structure of CN and CNCA. (**c**) PL spectra, (**d**) TR-PL spectra of CN/CF and CNCA/CF. (**e**) Photocurrent response, (**f**) Nyquist plots of CN and CNCA.

To assess the optical properties of catalysts, the UV–vis diffuse reflectance spectra were measured. Compare to CN/CF, CNCA/CF has a stronger light absorption capability (Figure S8). The absorption edge appears at around 400 nm due to being deposited on the carbon fiber substrate. Correspondingly, without substrate interference, CNCA powder also exhibited much stronger light absorption than pristine CN and the absorption edge red-shifts remarkably (Figure 3a). The incorporation of cinnamylidene groups resulted in a color transition of the powder from pale yellow to orange. This extended light absorption originated from a stronger n → π* electronic transition and *π* → π* transition. In particular, the n → π* electronic transition is thought to be caused by the extended conjugated structure of cinnamylidene groups. Thus, this result demonstrates that CNCA can potentially improve its solar energy utilization efficiency to drive photocatalytic H_2_O_2_ evolution process. The bandgap value of the catalysts was calculated by the Kubelka–Munk function (Figure S9) and narrowed from 2.68 eV (CN) to 2.39 eV (CNCA). Mott–Schottky curve was tested at three different frequencies (1000, 2000 and 3000 Hz) to obtain the flat-band potentials (Figure S10). According to the above results, the conduction band (CB) positions were −0.43 V for CN and −0.55 V for CNCA, with the corresponding band structures are shown in Figure 3b. The CB positions of both CN and CNCA are located above the reduction potentials of O_2_/·O_2_^−^, which favors 2e ORR pathway for H_2_O_2_ synthesis [34,35,36]. In the pure water system, the 4e- water oxidation process following the half-reaction (2H_2_O + 4h^+^ → O_2_ + 4H^+^) represents a thermodynamically accessible pathway for photogenerated hole consumption. The valence band positions of CN and CNCA are more positive than the potential of H_2_O/O_2_, satisfying the thermodynamic requirement for this reaction.

To further evaluate charge carrier dynamics, photoluminescence (PL) spectra show that CNCA/CF exhibits significantly reduced emission intensity (Figure 3c). Time-resolved fluorescence decay spectroscopy (Figure 3d) reveals the average carrier lifetime for CNCA/CF (1.37 ns) is shorter than pristine CN/CF (2.03 ns) (Table S1), indicating that CNCA possesses superior photogenerated carrier separation efficiency [18,19]. The effective electron migration efficiency of CNCA might be resulted from the extended conjugation structure. Moreover, electrochemical measurement was conducted to investigate the photogenerated carrier separation efficiency. Compared to CN, CNCA have a clearly greater transient photocurrent response (Figure 3e), further confirming that the incorporation of cinnamylidene groups enhances light absorption and promotes charge carrier generation [37]. The conjugated structure formed by the double bond and benzene ring facilitates charge diffusion. Electrochemical impedance spectroscopy (EIS) reveals that CNCA has a smaller semicircular arc than that of CN (Figure 3f, Table S2), validating that it has lower charge transfer resistance [38]. This result stems from the conjugated structure serving as an electron-transfer bridge to promote carrier migration. Collectively, these results confirm that grafting cinnamylidene groups is conducive to accelerate charge migration of CN.

**Figure 4:**
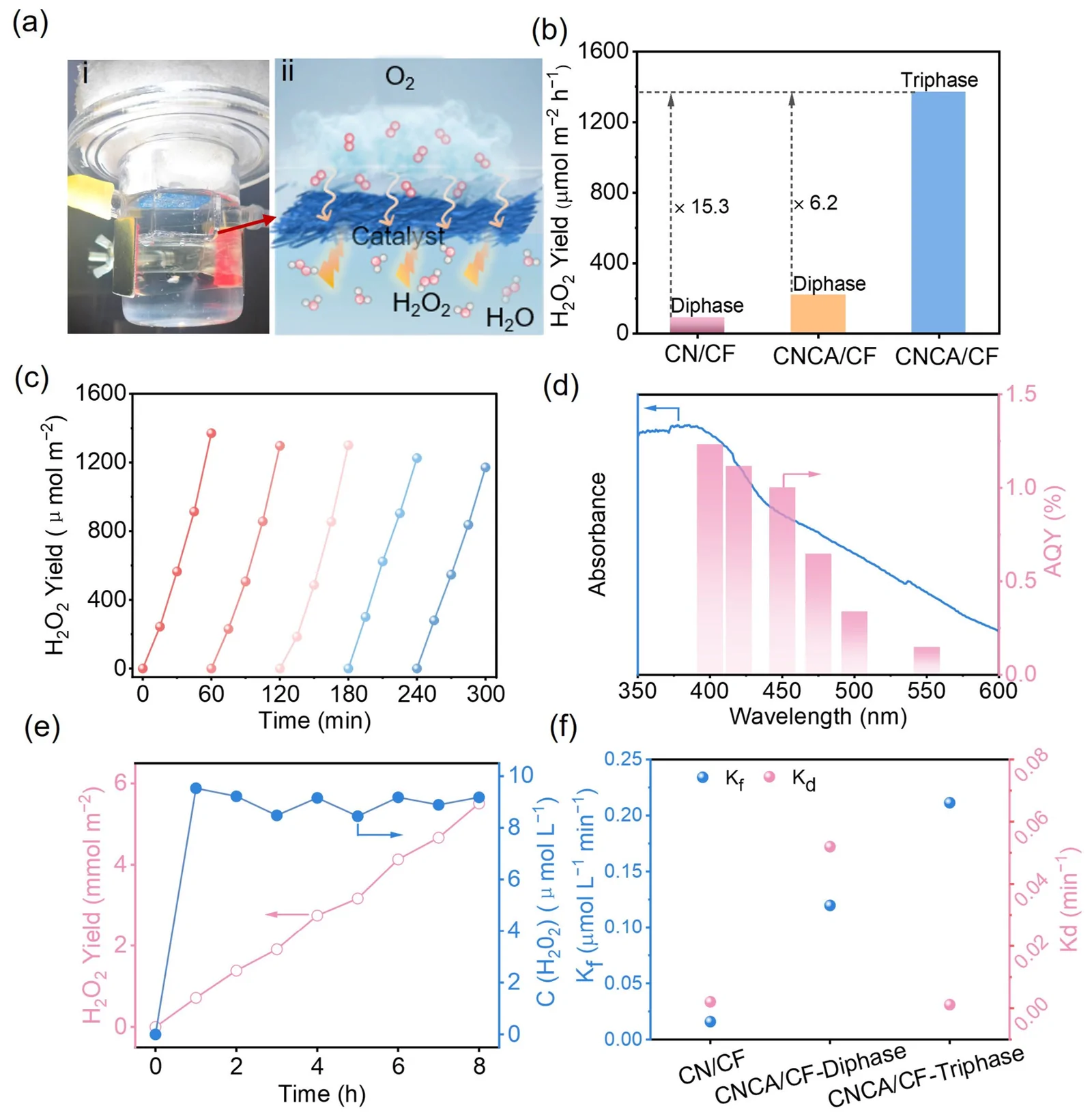
Photocatalytic H_2_O_2_ production performance. (**a(i)**) Photograph of CNCA/CF floated on water, (**a(ii)**) schematic illustration of triphase interface. (**b**) Photocatalytic H_2_O_2_ production by CN/CF in diphase system and CNCA/CF (in diphase and triphase system, respectively) in pure water. (**c**) Cycled H_2_O_2_ generation by CNCA/CF in the triphase system. (**d**) The *AQY* of CNCA/CF. (**e**) Time-dependence of flow photocatalytic H_2_O_2_ production on the CNCA/CF. (**f**) The formation rate constants (*K_f_*) and decomposition rate constants (*K_d_*).

The performance of H_2_O_2_ generation by hydrophobic CNCA/CF was studied by floating the catalyst on the water surface irradiated under the bottom, forming a triphase interface (Figure 4a(i) and Figure S11). The upper surface was exposed to an O_2_ atmosphere while the lower surface contacts the water phase, thus establishing a gas–solid–liquid triphase system (Figure 4a(ii)). In this triphase system, the O_2_ diffusion rate across the porous carbon fiber surface is near to that in air, enabling rapid O_2_ transport through the carbon fiber to the catalytic interface. In contrast, for the diphase system, the entire interface is immersed within aqueous phase. The poor solubility and sluggish diffusion of oxygen in water restricts oxygen transfer at the catalyst surface. Therefore, this unique floating configuration enhances mass transfer of O_2_ molecules compared to diphase systems. For comparison, CNCA/CF-diphase was acquired by pretreating the porous matrix with dimethyl sulfoxide (DMSO).

Photocatalytic performance revealed that CNCA/CF in the triphase system exhibits a remarkable H_2_O_2_ production rate of 1370 μmol m^−2^ h^−1^ (462.8 µmol g^−1^ h^−1^), representing almost 6.2 and 15.3-fold enhancements compared to CNCA/CF and CN/CF in diphase systems, respectively (Figure 4b, Figures S12 and S13). The enhanced H_2_O_2_ yield in the triphase system demonstrates that the hydrophobic CNCA/CF network effectively facilitates rapid O_2_ supply during photocatalytic H_2_O_2_ evolution. In addition, for the diphase system, the performance of CNCA/CF outperforms CN/CF thanks to its superior light harvesting and charge separation capabilities, which are facilitated by the extended conjugation system of the cinnamylidene groups. A slight decrease in the H_2_O_2_ generation was found after five consecutive tests, while the catalyst still maintained high activity (Figure 4c). As shown in Figure 4d, the apparent quantum yield (*AQY*) of CNCA/CF tracks well with the corresponding DRS spectral profile. The results reveal that the *AQY* of CNCA/CF at 550 nm can maintain 0.15%, indicating that an absorption edge extends into the visible region (Figure 4d). Despite the relatively low surface area of the CNCA/CF fiber network, the *AQY* still retains 1.23% at 400 nm, indicating high H_2_O_2_ production activity. Moreover, CNCA/CF achieves continuous H_2_O_2_ accumulation over 8 h applied in a continuous-flow reactor, with a linearly increasing concentration reaching 9.18 μmol L^−1^ (Figure 4e). To evaluate the stability of CNCA/CF, structural and morphological characterizations including XRD, FTIR and SEM were performed. The results revealed that negligible change after use (Figures S14 and S15), indicating a robust structure.

Notably, the detected H_2_O_2_ concentration is contingent upon the competitive formation and decomposition of H_2_O_2_, following the kinetics formula: [H_2_O_2_] = (*K_f_*/*K_d_*){1−exp(−*K_d_*t)}, with *K_f_* and *K_d_* being the zero-order and first-order kinetic constant, respectively) [39]. Based on this kinetics formula, *K_f_* and *K_d_* for CN/CF, CNCA/CF-diphase and CNCA/CF-triphase were presented in Figure 4f. For all catalysts, *K_f_* is one order of magnitude higher than *K_d_*, confirming their capability to efficiently produce H_2_O_2_. Compared to the diphase system of CNCA/CF, the triphase system displayed a 1.8-fold increase in *K_f_* and a marked suppression of *K_d_*, mainly due to the rapid oxygen supply to the catalyst active sites at the triphase interface. Notably, the *K_f_* of CNCA/CF-diphase is seven times higher compared to CN/CF, owing to the conjugated cinnamylidene groups grafted on the CN surface that promote electron migration to enhance O_2_ activation, thereby improving surface reaction kinetics. Collectively, these results demonstrate that the superior H_2_O_2_ accumulation in the triphase CNCA/CF interface benefits from both enhanced intrinsic catalytic activity and accelerated O_2_ supply, which accelerated H_2_O_2_ formation and suppress its decomposition.

**Figure 5:**
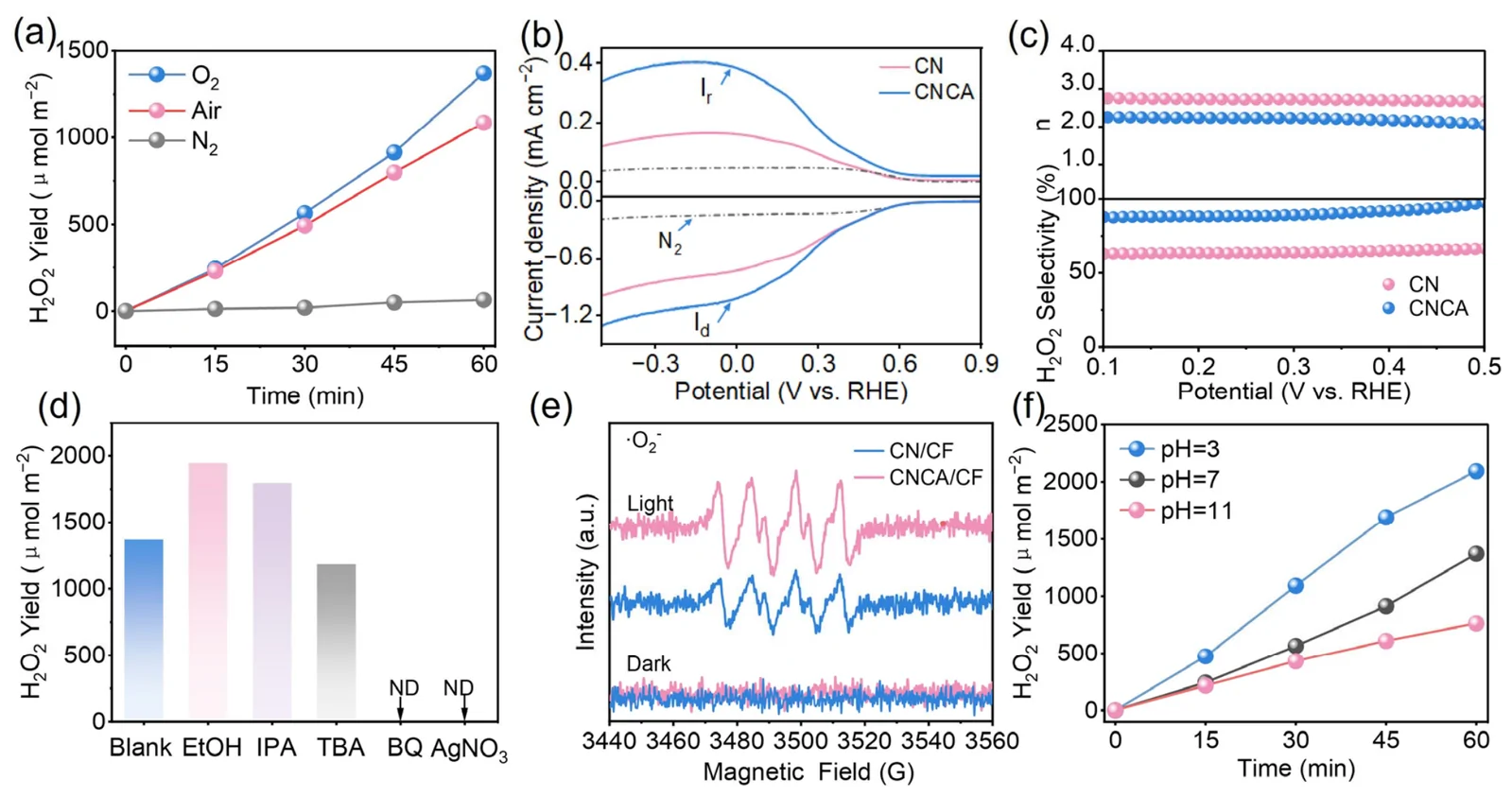
Photocatalytic H_2_O_2_ production mechanism investigation. (**a**) H_2_O_2_ yields of CNCA/CF in different atmosphere. (**b**) Linear-sweep RRDE voltammograms over CN and CNCA at 1600 rpm in KOH solution (pH = 13). (**c**) H_2_O_2_ selectivity and average electron transfer number. (**d**) Trapping experiments with dosing different scavengers in pure water for H_2_O_2_ production. (**e**) EPR spectra to detect ·O_2_^−^ radicals over CN/CF and CNCA/CF with and without illumination. (**f**) H_2_O_2_ yields in different pH solution.

To elucidate the reaction mechanism, H_2_O_2_ production was performed in different atmospheres. As shown in Figure 5a, the H_2_O_2_ yield in O_2_ atmosphere was higher than in air (1085 μmol m^−2^ h^−1^). It was noted that H_2_O_2_ yield was significantly suppressed in N_2_ (64 μmol m^−2^ h^−1^). The results of these control experiments indicate that O_2_ plays a critical role for photocatalytic H_2_O_2_ generation. The rotating ring-disk electrode (RRDE) was employed to analyze the ORR pathway in the O_2_-saturated KOH solution (0.1 M, pH = 13) [38]. As observed in Figure 5b, ORR currents of CNCA and CN start ~0.62 V vs. RHE in O_2_-saturated solution, with a saturation plateau at −0.2~−0.5 V vs. RHE. The rising ring current (*I_r_*) coupled with disk current (*I_d_*) caused by H_2_O_2_ oxidation, with H_2_O_2_ formed on the disk and then diffusing to the ring (Figure 5b). Notably, CNCA shows slightly higher I_d_ than CN, the *I_r_* of CNCA is more than two times that of CN (Figure 5b). The electron transfer numbers (n) for CNCA and CN correspond to 2.3 and 2.7, respectively (Figure 5c), suggesting that CNCA preferentially follows the 2e^−^ ORR pathway. Correspondingly, CNCA exhibits a H_2_O_2_ selectivity of 87%, much higher than that of CN (64%) (Figure 5c), which is consistent with the catalytic activity. The higher H_2_O_2_ selectivity of CNCA primarily originates from the introduction of conjugated cinnamylidene groups to promote electron migration.

To reveal the H_2_O_2_ synthesis pathway, radical trapping experiments were carried out [40]. H_2_O_2_ yield was increased to 1.95 mmol m^−2^ h^−1^ after introducing ethanol (EtOH) as a hole scavenger in Figure 5d. In contrast, after dosing the electron scavenger (AgNO_3_) and the ·O_2_^−^ scavenger (1,4-benzoquinone, BQ), H_2_O_2_ production was completely inhibited. Meanwhile, after dosing the ·OH scavenger (tert-butanol, TBA), H_2_O_2_ yield only a slight decrease (Figure 5d). These results confirm that photogenerated electrons are essential for H_2_O_2_ generation. Meanwhile, ·O_2_^−^ acts as a vital intermediate in the 2e ORR process. To further confirm the presence of ·O_2_**^−^**, Electron paramagnetic resonance (EPR) was conducted with DMPO as a trapping agent [41]. Clearly, a strong DMPO-·O_2_^−^ signal was observed after irradiation, whereas no signal was detected in the dark (Figure 5e). Moreover, the DMPO-·O_2_^−^ signal generated by CNCA/CF is much stronger than that of CN. This result agrees well with the enhanced photocatalytic H_2_O_2_ generation activity of CNCA and ·O_2_^−^ is identified as a key intermediate during the photosynthesis of H_2_O_2_. Additionally, the H_2_O_2_ yield was the highest in acidic solution, compared with neutral and alkaline conditions, indicating that protons also play an important role in this process (Figure 5f).

## 3. Materials and Methods

### 3.1. Preparation of CN/CF

A carbon nitride nanolayer was in-situ grown onto carbon felt by the chemical vapor-assisted process. Typically, melamine (0.5 g, Aladdin, Shanghai, China) was loaded at the bottom of a glass tube, while carbon felt (2.0 cm × 2.0 cm × 0.3 cm, Inner Mongolia, China) was suspended in the tube without touching the melamine. The tube was covered with aluminum foil, followed by heating at 5 °C min^−1^ up to 550 °C and holding for 4 h in air. Sublimed melamine was coated on the carbon fiber and polymerized into a dense CN nanolayer. After cooling down naturally, the CN/CF was collected. To obtain CN powder, 5 g melamine was put into a crucible and then calcined at 550 °C for 4 h in air atmosphere.

### 3.2. Preparation of Hydrophobic CNCA/CF

The preformed CN/CF was positioned in a quartz tube with cinnamaldehyde (1 mL, Aladdin, Shanghai, China) at the bottom. Then, the quartz tube was purged with N_2_ to expel air and rapidly sealed using aluminum foil. The sealed tube was transferred into the tube furnace and N_2_ flow was maintained for 10 min to remove residual air. Subsequently, it was heated to 300 °C for 1 h in N_2_ atmosphere. Then, hydrophobic CNCA/CF was collected after a Schiff-base reaction (Figure S1). The CNCA powder was prepared following same synthetic procedure of CNCA/CF, without using carbon felt substrate. A total of 0.1 g pristine CN was put in the middle of the tube, while 1.0 mL cinnamaldehyde was placed at the bottom. After sealing tube, the CNCA powder was synthesized by calcinating.

### 3.3. Materials Characterization

The characterization methods and instruments are provided in the Supplementary Information (SI).

### 3.4. Photocatalytic Production of H_2_O_2_

Photocatalytic H_2_O_2_ production was performed by placing a carbon fiber network material in a quartz cup reactor containing pure water. The hydrophobic CNCA/CF can float on the water surface to form a triphase interface, while the hydrophilic CN/CF sinks to the bottom of the water. The quartz cup was irradiated from the bottom using a Xe lamp (300 W, China Education Au-light, CEL-HXUV300E-T3, Beijing, China) and light intensity at the reactor surface was set to 200 mW cm^−2^ (the distance between the Xe lamp and the surface of the reactor was 29.5 cm), measured using a radiometer (Model FZ-A Perfect Light, Beijing, China). The photocatalytic performance test was performed with a catalyst (an illuminated area of 1 × 1 cm^2^) in 10 mL pure water and O_2_ flow rate was maintained at 30 mL min^−1^. The H_2_O_2_ production rate (μmol m^−2^ h^−1^) was calculated using the following formula:

$$\mathrm{Rate}=\frac{C\times V}{A\times t}$$

where *C* is the measured H_2_O_2_ concentration (μmol L^−1^), *V* is the volume of the reaction solution (L), *A* is the illuminated area of the catalyst (m^2^) and *t* is the irradiation time (h).

For comparison, the pretreated CNCA/CF was obtained to construct a diphase interface, which was wetted with the aprotic solvent DMSO and then rinsed with a large amount of water to render it hydrophilic. O_2_ was continuously bubbled on the surface of catalyst in the triphase system, while O_2_ was continuously sparged into the water in the diphase system. O_2_ flow rate was continuously maintained at 30 mL min^−1^. The reaction was carried out in a quartz cup reactor equipped with an outer circulating cooling jacket for temperature control.

The geometric-area-based rate can be converted to mass-normalized activity using the catalyst mass and geometric area of carbon felt:

$$R_{m}=R_{g}\times \frac{A_{g}}{m_{\mathrm{catalyst}}}$$

where *R_g_* is the geometric-area-based H_2_O_2_ production rate, *A_g_* is the irradiated geometric area of catalyst on carbon felt and *m_catalyst_* is the mass of the catalyst loaded on carbon felt.

The concentration of H_2_O_2_ was measured by a UV–vis spectrophotometer in accordance with HRP/TMB colorimetric method (HRP, the horseradish peroxidase; TMB, 3,3′,5,5′-tetramethyl benzidine; Sinopharm Chemical Reagent Co., Ltd., Shanghai, China). A detection mixture (1 mL) was composed of 0.5 mL HRP (50 µg mL^−1^) and 0.5 mL TMB (100 µmol L^−1^) dispersed in acetate buffer (0.1 M, pH3.5). This mixture was blended with reaction solution (0.2 mL). With HRP acting as a catalyst, the colorless TMB was oxidized by H_2_O_2_, producing the blue oxidation product TMB-O_x_. Finally, the H_2_O_2_ concentration was quantified by the absorbance of TMB-O_x_ solution at 652 nm, coupled with a standard calibration curve that connects absorbance to H_2_O_2_ concentration. The detection wavelength range was set from 400 nm to 800 nm and the corresponding calibration curve was constructed within this range.

The apparent quantum yield (*AQY*) was measured to evaluate the photocatalytic H_2_O_2_ generation performance. Monochromatic light with different wavelengths was acquired using a CEL-HXUV300E-T3 light source fitted with a series of bandpass optical filters. The light intensity was maintained at 200 mW cm^−2^ before installing the bandpass filter and measured using a radiometer (Model FZ-A Perfect Light, China), with the irradiated area kept at 1 cm^2^. The *AQY* was quantified via the equation:

*AQY* (%) = 2 × *N*(*H*_2_*O*_2_)/*N_photons_* × 100%

where *N*(*H*_2_*O*_2_) is the molar amount of produced H_2_O_2_. *N_photons_* is the total photon number; the *N_photons_* was calculated by *N_photons_* = *Ptλ*/*NAhc* = *IStλ*/*NAhc*. The constants and variables are defined as: Planck constant *h* = 6.63 × 10^−34^ J s; the Avogadro constant *N_A_* = 6.02 × 10^23^ mol^−1^; speed of light *c* = 3 × 10^8^ m s^−1^; *I*, *S*, *t* and *λ* represent light irradiation intensity (mW cm^−2^), irradiated area (cm^2^), photoreaction time (s) and the wavelength of monochromatic light (nm), respectively.

## 4. Conclusions

In summary, a hydrophobic CNCA/CF photocatalyst was successfully developed by grafting cinnamaldehyde onto CN nanolayers via Schiff base reaction. Owing to its hydrophobic surface, CNCA/CF can float on water surface and construct an effective gas–solid–liquid triphase interface, which facilitates rapid O_2_ supply to the catalytic surface. The incorporation of cinnamylidene groups can broaden the light absorption range and accelerate the migration of photogenerated charges by extending the conjugated structure. Accordingly, CNCA/CF in the triphase system exhibits a high H_2_O_2_ yield of 1370 μmol m^−2^ h^−1^. This H_2_O_2_ evolution rate surpasses the diphase CNCA/CF (6.2 times) and pristine CN/CF (15.3 times), respectively. Mechanistic investigations verify that H_2_O_2_ generation undergoes an indirect 2e ORR pathway (O_2_ →·O_2_^−^ → H_2_O_2_). This work underscores that combining triphase interface engineering with surface functionalization by extended conjugated structure is an effective strategy to boost photocatalytic H_2_O_2_ production. It also provides a rational design guideline for the development of high-performance floatable photocatalysts.

## Data Availability

The original contributions presented in this study are included in the article/Supplementary Material. Further inquiries can be directed to the corresponding authors.

## Supplementary Materials

The following supporting information can be downloaded at: https://www.mdpi.com/article/10.3390/molecules31203770/s1, Figure S1: A schematic illustration of Schiff-base reaction between CN and cinnamaldehydes. Figure S2: Photograph of pristine carbon felt. Figure S3: (a) Photograph of hydrophobic CNCA/CF floating on the water surface. (b) Photograph of CN/CF submerged in water. Figure S4: SEM of CF. Figure S5: (a,b) EDS element mapping and (c) Element distribution of CNCA/CF. Figure S6: (a) XRD of CF. (b) XRD of CN and CNCA powder. (c) Full-range FTIR spectra of CF, CN/CF and CNCA/CF. Figure S7: (a) The high-resolution N 1s spectra. (b) The high-resolution O 1s spectra. (c)The XPS survey spectra of CN/CF and CNCA/CF. (d) Atomic percentage of CN/CF and CNCA/CF obtained from XPS survey. Figure S8: UV-vis DRS of CN/CF and CNCA/CF. Figure S9: Corresponding plots of the converted Kubelka–Munk function for CN and CNCA. Figure S10: Mott–Schottky curves of (a) CN and (b) CNCA. Figure S11: The Photograph of photocatalytic production of H_2_O_2_. Figure S12: (a) The UV-vis absorptions of standard solutions and (b) The standard absorption–concentration plots for H_2_O_2_ concentration titration. Figure S13: Photocatalytic H_2_O_2_ generation by CN/CF in diphase system, and CNCA/CF (in diphase and triphase system, respectively) in pure water. Figure S14: (a) XRD and (b) FTIR of CNCA/CF before and after use. Figure S15: Cross-sectional SEM of CNCA/CF after cycling test. Table S1: Charge carrier lifetime obtained from the time-resolved fluorescence spectra. Table S2: The Quantitative values for R_s_, R_ct_, and C of CN and CNCA.
