# Supplementary material for: Boosted H2O2 Photosynthesis via Synergistic Tuning Electronic Structure and Interfacial O2 Transfer on Floatable Carbon Nitride Networks
Source: Molecules. 2026 Oct 20;31(20):3770. doi: 10.3390/molecules31203770 (PMC13707579; doi:10.3390/molecules31203770)
Supplement: Supplementary file 1 [file molecules-31-03770-s001.zip › molecules-4625940-supplementary.pdf]

Supporting Information

Boosted H_2O_2 Photosynthesis via Synergistic Tuning Electronic Structure and Interfacial O_2 Transfer on Floatable Carbon Nitride Networks

Xiaoying Peng[1], Zhangsong Jiang[1], Fanghua Liu[1], Bingzhi Xiang[1], jielu Zhu[1], Jie Lin[1], Bo Wang[1], Junwei Song[1], Kai Yang[2], Guiming Peng[2]**

[1] Research Center of Advanced Materials and Green Energy, Jiangxi Institute of Technology, Nanchang, 330098, China

[2] School of Chemistry and Chemical Engineering, Jiangxi University of Science and Technology, Ganzhou, 341000, China

[3] College of Chemistry and Materials, Key Laboratory of Fluorine and Silicon for Energy Materials and Chemistry of Ministry of Education, Jiangxi Normal University, Nanchang, 330022, China

E-mail: yangkai@jxust.edu.cn (K.Y.), guiming@jxnu.edu.cn (G.P.)

Materials characterization

The morphologies were characterized by a scanning electron microscope (SEM, ZEISS Sigma 300 FE-SEM, Germany). X-ray diffraction (XRD) patterns were obtained on a Bruker D8 Advance diffractometer using Cu Kα X-ray radiation (λ = 1.54 Å) at room temperature. X-ray photoelectron spectroscopy (XPS) spectra were conducted on an X-ray photoelectron spectrometer (Thermo Scientific Nexsa, USA) with Mg Kα as the source and the C 1s peak at 284.6 eV as an internal standard. FTIR spectra analysis was performed using Fourier transform infrared spectrometer Nicolet 6700 (USA). Photoluminescence (PL) spectra were recorded with a steady-state transient fluorescence spectrometer (FLS980, UK). Solid ^{13}C NMR results were collected on Bruker 400M. UV-Vis diffuse reflectance spectra (DRS) were acquired with a UV-Vis-NIR spectrophotometer (Shimadzu, UV-2600i, Japan) equipped with an integrating sphere. The electron paramagnetic resonance (EPR) measurement was conducted to detect the radicals with DMPO as the radical trapping reagent using an A300 EPR

(Germany) spectrometer. Contact angle measurements (SL200KS, Shanghai, China) were used to evaluate the hydrophobicity of CN/CF and CNCA/CF.

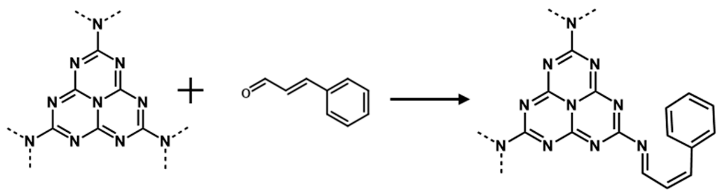

Figure S1. A schematic illustration of Schiff-base reaction between CN and cinnamaldehydes.

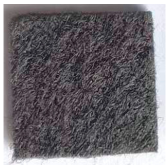

Figure S2. Photograph of pristine carbon felt.

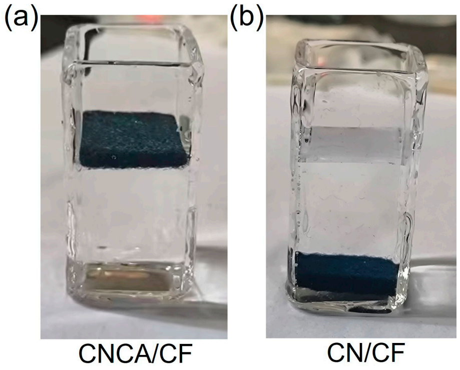

Figure S3. (a) Photograph of hydrophobic CNCA/CF floating on the water surface. (b) Photograph of CN/CF submerged in water.

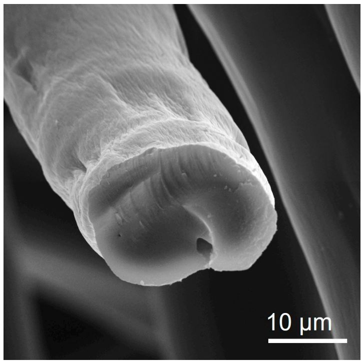

Figure S4. SEM of CF.

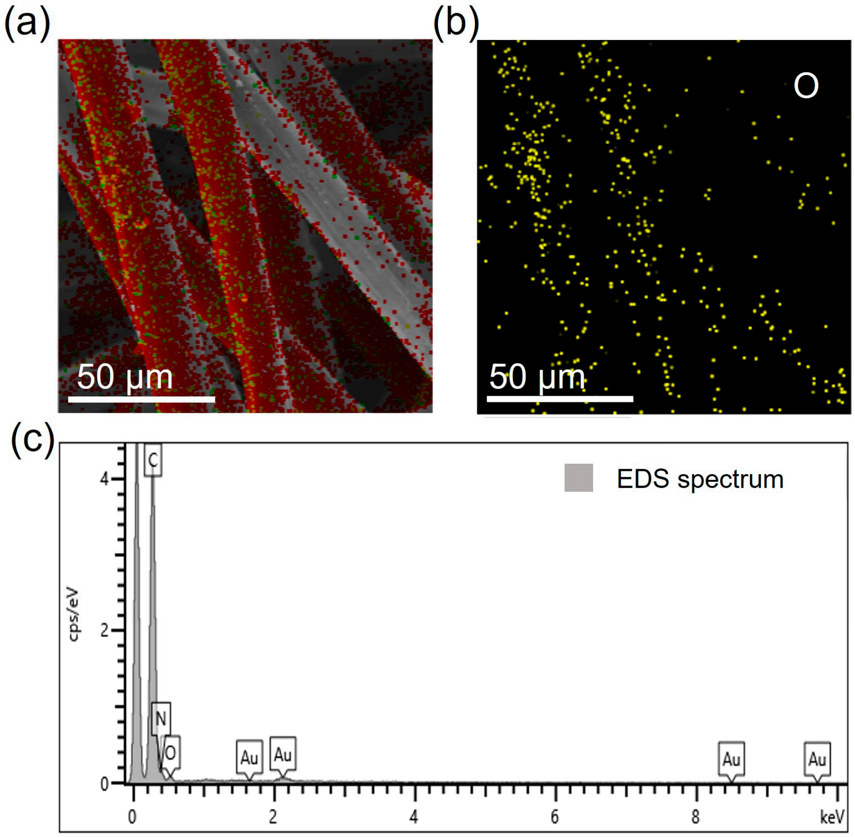

Figure S5. (a, b) EDS element mapping and (c) Element distribution of CNCA/CF.

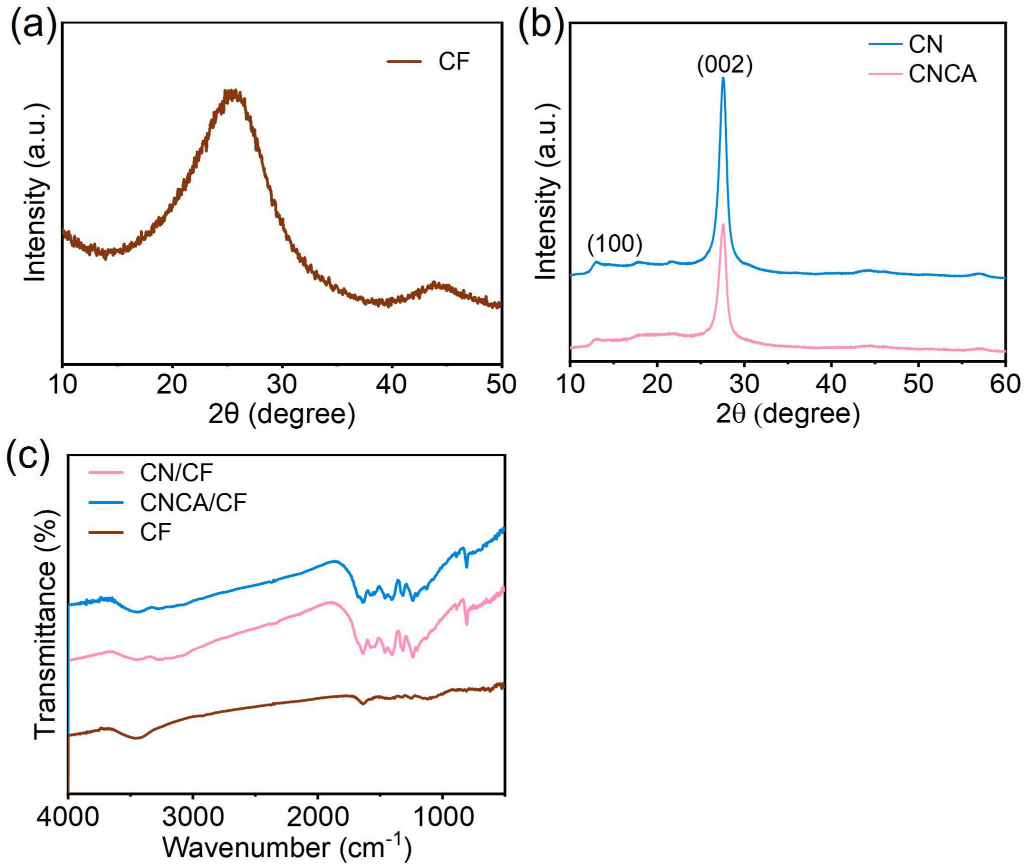

Figure S6. (a) XRD of CF. (b) XRD of CN and CNCA powder. (c) Full-range FTIR spectra of CF, CN/CF and CNCA/CF.

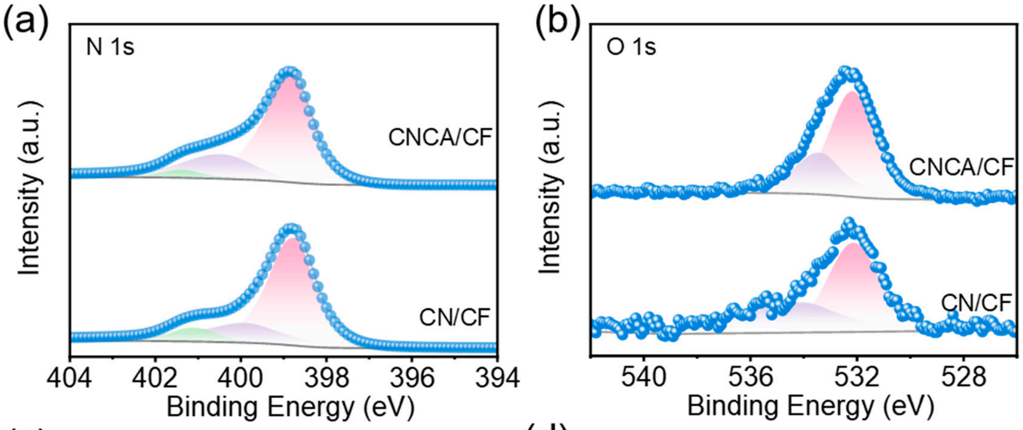

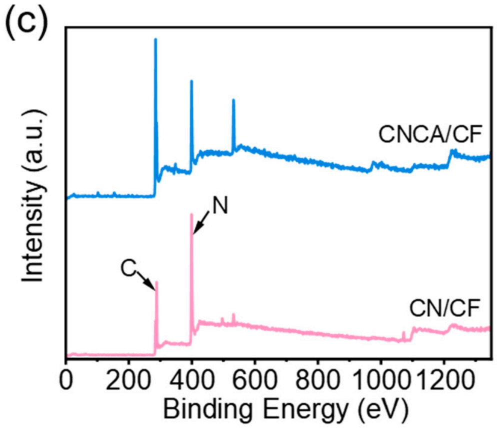

(d)

Atomic %	CN/CF	CNCA/ CF
C	46.88	67.79
N	48.72	22.10
O	4.40	10.11
C/N	0.96	3.07

Figure S7. (a) The high-resolution N 1s spectra. (b) The high-resolution O 1s spectra. (c)The XPS survey spectra of CN/CF and CNCA/CF. (d) Atomic percentage of CN/CF and CNCA/CF obtained from XPS survey.

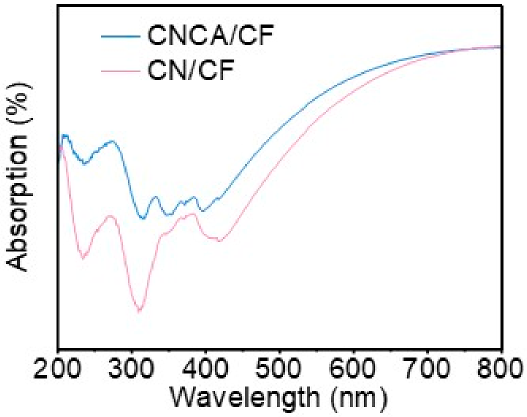

Figure S8. UV-vis DRS of CN/CF and CNCA/CF.

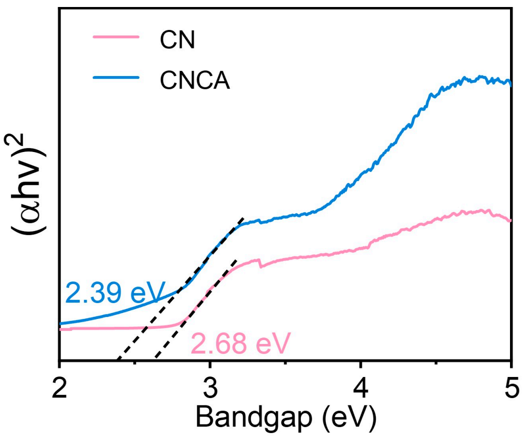

Figure S9. Corresponding plots of the converted Kubelka–Munk function for CN and CNCA.

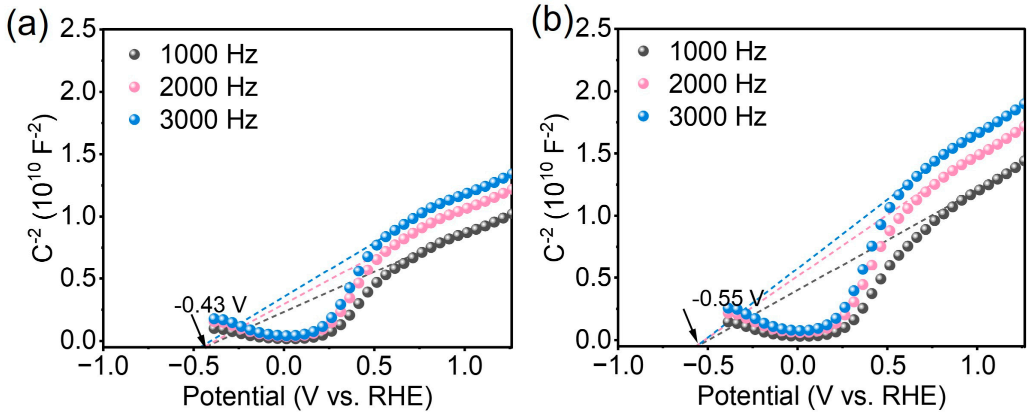

Figure S10. Mott–Schottky curves of (a) CN and (b) CNCA.

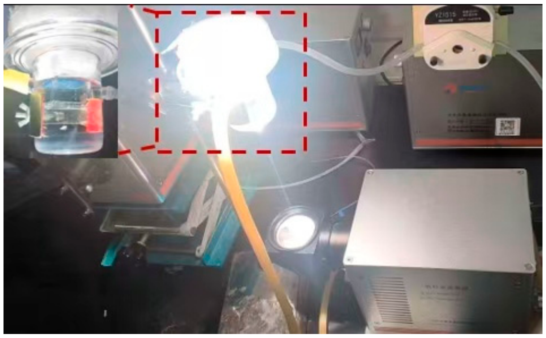

Figure S11. The Photograph of photocatalytic production of H_2O_2.

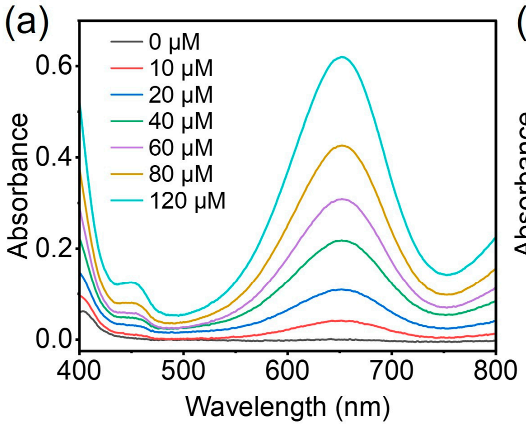

Figure S12. (a) The UV-vis absorptions of standard solutions and (b) The standard absorption–concentration plots for H_2O_2 concentration titration.

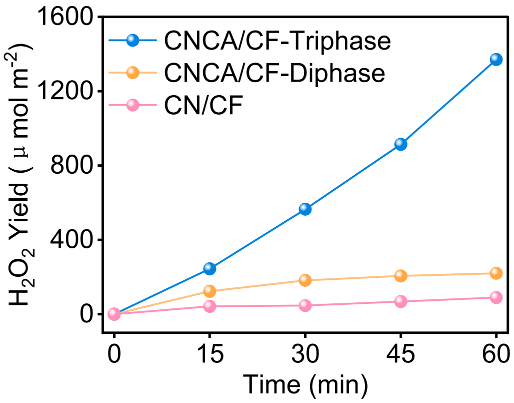

Figure S13. Photocatalytic H_2O_2 generation by CN/CF in diphase system, and CNCA/CF (in diphase and triphase system, respectively) in pure water.

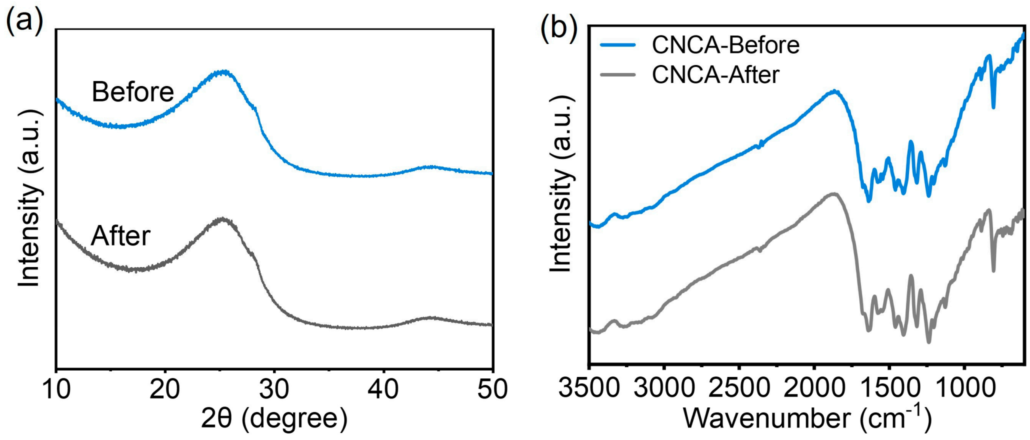

Figure S14. (a) XRD and (b) FTIR of CNCA/CF before and after use.

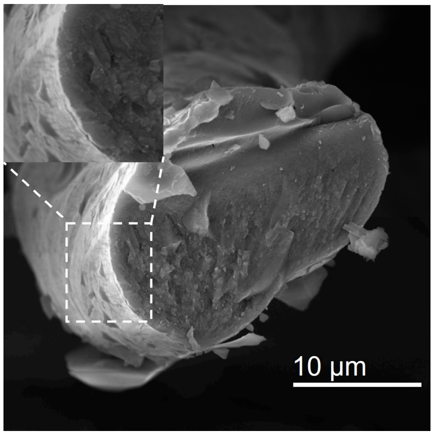

Figure S15. Cross-sectional SEM of CNCA/CF after cycling test.

Table S1: Charge carrier lifetime obtained from the time-resolved fluorescence spectra.

Samples	τ_1	A_1	τ_2	A_2	τ (ns)
CN/CF	0.48	0.018	3.36	0.003	2.03
CNCA/CF	1.37	0.016	-	-	1.37

Table S2. The Quantitative values for R_s, R_{ct}, and C of CN and CNCA.

Samples	R_s (Ω)	R_{ct} (Ω)	C (F cm^{-2})
CN/CF	28.05	1.525 e^{6}	1.434 e^{-5}
CNCA/CF	30.98	7.862 e^{5}	1.243 e^{-5}
